# Second-to-Fourth Digit Ratio and Impulsivity: A Comparison between Offenders and Nonoffenders

**DOI:** 10.1371/journal.pone.0047140

**Published:** 2012-10-17

**Authors:** Yaniv Hanoch, Michaela Gummerum, Jonathan Rolison

**Affiliations:** 1 School of Psychology, Cognition Institute, University of Plymouth, Plymouth, United Kingdom; 2 School of Psychology, Queen's University Belfast, Belfast, United Kingdom; George Mason University/Krasnow Institute for Advanced Study, United States of America

## Abstract

Personality characteristics, particularly impulsive tendencies, have long been conceived as the primary culprit in delinquent behavior. One crucial question to emerge from this line of work is whether impulsivity has a biological basis. To test this possibility, 44 male offenders and 46 nonoffenders completed the Eysenck Impulsivity Questionnaire, and had their 2D∶4D ratio measured. Offenders exhibited smaller right hand digit ratio measurements compared to non-offenders, but higher impulsivity scores. Both impulsivity and 2D∶4D ratio measurements significantly predicted criminality (offenders vs. nonoffenders). Controlling for education level, the 2D∶4D ratio measurements had remained a significant predictor of criminality, while impulsivity scores no longer predicted criminality significantly. Our data, thus, indicates that impulsivity but not 2D∶4D ratio measurements relate to educational attainment. As offenders varied in their number of previous convictions and the nature of their individual crimes, we also tested for differences in 2D∶4D ratio and impulsivity among offenders. Number of previous convictions did not correlate significantly with the 2D∶4D ratio measurements or impulsivity scores. Our study established a link between a biological marker and impulsivity among offenders (and lack thereof among non-offenders), which emphasise the importance of studying the relationship between biological markers, impulsivity and criminal behavior.

## Introduction

With record number of people in US prisons and an estimated annual cost of crime exceeding $1 trillion [1], it is no wonder that researchers have been interested in the underlying mechanisms involved in criminality. From the 19^th^ century to the present day, personality characteristics—primarily impulsivity—have been conceived as one of the primary culprits in delinquent behavior [2]. The influential General Theory of Crime [3] emphasises that offending is a function of opportunity and impulsivity—usually conceived as quick processing of information, novelty seeking, and inability to delay gratification [4]—with a large body of evidence supporting a connection between impulsivity and criminality [5]. Prisons, likewise, have been relying on impulsivity measures to evaluate the success of behavioral change programs [6].

The search for a biological basis for personality characteristics, especially impulsivity, has a long tradition [7]. As one of the most common and easy to measure indicators of prenatal androgens exposure, researchers have used 2D∶4D digit ratio (the ratio of the second finger over the fourth ring finger length) [8], [9] as a means to gauge the biological basis of personality. One interesting strand of research has explored the link between attention deficit/hyperactivity disorder (ADHD). One study [10] reported a link between 2D∶4D digit ratio and parents' and teachers' rating of ADHD symptoms among boys but not girls. Another study among young children [11], however, found no similar relationship between 2D∶4D and ADHD symptoms. Examining adult populations [12], investigators have revealed a more complex picture. Among females, the greater left hand 2D∶4D ratio are associated with greater ADHD (inattentive as well as hyperactive-impulsive measures) symptoms; possibly due to small sample size, however, the authors did not report similar results among males.

A number of investigators have also explored a possible association between 2D∶4D ratio and criminality. Focusing on college students, an interesting investigation [13] has shown that 2D∶4D ratio correlate with psychopathy and callous affect. Investigating incarcerated methamphetamine users, others [14] have found that greater prenatal testosterone exposure, as indicated by 2D∶4D ratio, is associated with increases in anger thinking and a reduction in cognitive flexibility, at least among individuals who have experienced physical abuse [14].

The above studies are among a growing body of literature examining the link between 2D∶4D ratio and individual differences measures. With regard to impulsivity and criminal behavior, Aluja and colleagues [15] reported a link between impulsivity and serotonin transporter gene polymorphisms among inmates. Researchers have also found that 2D∶4D digit ratio is associated with males' aggressiveness [16], risk taking [17], [18] and sensation seeking [19]. Whether similar a relationship exists between 2D∶4D ratio and impulsivity among offenders and nonoffenders, however, is unknown. As such, our study could shed light on biological determinants to criminal behavior as well as impulsivity. In probably the first study to examine offenders' and nonoffenders' impulsivity and 2D∶4D ratio, we compared impulsive tendencies between the two groups, and examined whether a link exists between their 2D∶4D ratio and criminality.

## Methods

### Participants and Procedure

Prior to data collection, ethical approval for the research protocol was provided by both the university and participating organization and all participants provided written informed consent. Participants were 44 male offenders aged 21–58 years (*M*_age_ = 39.12; *SD* = 8.65) who were within 16 weeks of their prison release. Theft/burglary (27/44; 61%) was the predominant reason for incarceration, followed by drug offences (23/44; 52%) and violence (20/44; 45%). The majority of offenders had more than ten previous convictions (30/44; 68%), and few had one to five (8/44; 18%) or between six and ten previous convictions (6/44; 14%).We compared the offenders with 46 nonoffenders—who were recruited from the community—of a similar age range (*M*_age_ = 31.30; *SD* = 9.72; range 19–57). Offending and nonoffending participants also indicated on a scale from 0 to 6 their highest educational attainment (0 = no education, 1 = key stage 1, 2 = key stage 2, 3 = GCSE, 4 = Diploma/vocational, 5 = A-level, 6 = degree) (Key stage 1 and key stage 2 refer to primary school. GCSE is an academic qualification awarded to those who have finished high school at 16 years of age, whereas A-level is equivalent to matriculating from high-school with the necessary requirements to attend university). Ten of the 44 (23%) offenders indicated that their highest educational attainment was the General Certificate of Secondary Attainment (GCSE), compared to 18 (39%) nonoffenders. Similarly, 9 (20%) of the nonoffenders and none of the offenders, indicated A-levels as their highest attainment. Nonoffenders were younger than the offenders, *t*(86) = 3.972, *p*<.001, and were better educated, Mann-Whitney *U* = 534, *p*<.001.

### Measures

Male offenders and nonoffenders completed the Eysenck Impulsivity Questionnaire [20], which has been extensively used with both offending and nonoffending populations and has been shown to have the best predictive value with regard to convictions and prison breaches of discipline [21]. To measure the 2D∶4D ratio, participants' right hand with their fingers together was scanned using a HP Scanjet 3800 with a resolution of 2400×4800 color dpi. As the majority of studies have used the right hand only, our study followed this procedure. Two independent raters, blind to the hypothesis, used a digital Vernier Caliper tool (to the precision of 0.01 mm) to measure the length of the second figure and the fourth ring finger (of the right hand only) from the ventral proximal crease to the tip of the digit on the scanned image. The 2D∶4D ratio measurement—which is thought to negatively correlate with prenatal testosterone exposure—was calculated separately for each participant by dividing the second finger measurement by the fourth finger measurement, using the mean measure of the two raters (internal consistency reliability, Cronbach's α = 0.996; *r* = .991).

## Results

**Figure 1:**
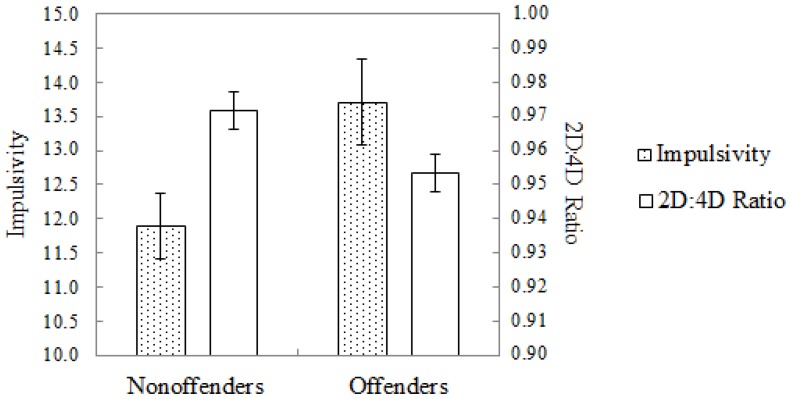
Mean group impulsivity scores and 2D∶4D right hand digit ratio measurements displayed separately for nonoffenders and offenders. Error bars represent 1 standard error around the mean.

Figure 1 displays the means (and standard errors) for the right hand digit ratio measurements, and impulsivity scores (summed across the 22 items). As expected, offenders exhibited smaller right hand digit ratio measurements compared to non-offenders, *t*(86) = 2.321, *p* = .023, but higher impulsivity scores, *t*(86) = 2.309, *p* = .023. A logistic regression analysis revealed that both impulsivity (OR = 1.15; 95% CI: 1.02, 1.29; *p* = .027) and 2D∶4D ratio measurements (OR<.01, 95% CI: <0.01, 0.21; *p* = .027) significantly predicted criminality (offenders vs. nonoffenders) when included as predictors in separate regression analyses. As the nonoffenders had an overall higher level of educational attainment than offenders, in a second step we also included education level as a covariate. Controlling for education level, the 2D∶4D ratio measurements remained a significant predictor of criminality (OR<.01, 95% CI: <0.01, 0.18; *p* = .027), while impulsivity scores no longer predicted criminality significantly (OR = 1.10, 95% CI: 0.96, 1.25; *p* = .163). Moreover, when impulsivity and 2D∶4D ratio measurements were included together in a final regression analysis with education level as a covariate, 2D∶4D ratio measurements (OR<.01, 95% CI: <0.01, 0.72; *p* = .045) and education level (OR = 0.55, 95% CI: 0.39, 0.77; *p*<.001), but not impulsivity scores (OR = 1.07, 95% CI: 0.94, 1.23; *p* = .323), predicted criminality.

This indicates that impulsivity but not 2D∶4D ratio measurements relate to educational attainment. Indeed, correlation analyses revealed that education level was negatively correlated with impulsivity (*r*(88) = −.251, *p* = .018) and unrelated to 2D∶4D ratio measurements (*r*(88) = .050, *p* = .644) across all participants, even though there were no significant correlations within the offending and nonoffending groups between education level and impulsivity (nonoffenders, *r*(46) = −.161, *p* = .286; offenders, *r*(42) = −.171, *p* = .280) and between education level and 2D∶4D ratio measurements (nonoffenders, *r*(46) = −.049, *p* = .748; offenders, *r*(42) = −.081, *p* = .612).

The correlation analyses above suggest that the 2D∶4D ratio may be linked to criminality through its effects on impulsivity and education, and that education could mediate effects of impulsivity on criminality. However, further correlation analysis revealed only a marginally significant correlation between 2D∶4D ratio measurements and impulsivity scores (*r*(88) = −.202, *p* = .059) across all participants, and within the groups, a significant correlation among offenders (*r*(42) = −.396, *p* = .009) but not nonoffenders (*r*(46) = .119, *p* = .432). Thus, 2D∶4D ratio appears to predict impulsivity only among offending individuals.

The offenders varied in their number of previous convictions and the nature of their individual crimes. For this reason, we also tested for differences in 2D∶4D ratio and impulsivity among offenders. Number of previous convictions did not correlate significantly with the 2D∶4D ratio measurements (*r*(42) = .039, *p* = .808) or impulsivity scores (*r*(42) = −.054, *p* = .735). Moreover, there were no significant mean group differences in 2D∶4D ratio measurements or impulsivity scores between those who had and those who had not been previously convicted for a drug related crime (2D∶4D, *t*(40) = 0.79, *p* = .434; impulsivity, *t*(40) = 0.63, *p* = .532), theft/burglary (2D∶4D, *t*(40) = 0.96, *p* = .345; impulsivity, *t*(40) = 0.84, *p* = .409), or violence (2D∶4D, *t*(40) = 0.15, *p* = .878; impulsivity, *t*(40) = 0.73, *p* = .473).

## Discussion

Researchers have postulated a link between impulsivity and crime, but have largely failed to examine whether offenders' impulsive tendencies might have a biological underpinning. Here, we bridged this lacuna by examining the link between 2D∶4D ratio and offenders' and nonoffenders' impulsive tendencies. We found that, even when controlling for education and impulsivity, 2D∶4D ratio significantly predicted criminality. Thus, our results nicely augment earlier investigations regarding the link between 2D∶4D ratio and criminality, such as the association between 2D∶4D ratio and psychopathy [12] as well as 2D∶4D ratio and anger rumination and cognitive flexibility among incarcerated methamphetamine users [13].

Consistent with other studies showing a relationship between testosterone levels and responses on the disinhibition scale of the sensation seeking scale among inmates [22], this study reveals a relationship between an important and well-studied biological marker [8], [9] and impulsivity among offenders. We did not find a link, however, between 2D∶4D ratio and impulsivity among our nonoffending sample. A number of possible factors can help explain this disparity between offenders and nonoffenders. Previous research has reported mixed findings concerning the relationship between 2D∶4D ratio and personality characteristics, such as risk preferences [23], [24], [25], sensation seeking [26] and even phenomenon such as ADHD [10], [11]. Whether 2D∶4D ratio is related to such personality characteristics might depend on sub-group examined, such as their ethnicity and gender. Particularly, 2D∶4D ratio is more likely to correlate with personality characteristics in homogeneous rather than heterogeneous subgroups [27], [28]. It is possible, therefore, that offenders represent a unique and more homogenous sub-group than non-offenders in terms of their current circumstances, life experiences, and social class.

Despite these encouraging results, we would caution against explaining criminal behavior by a single (biological or environmental) factor. Furthermore, researchers [29] have shown that 2D∶4D ratio is highly heritable. This might indicate that individual differences associated with 2D∶4D ratio could stem from heritable factors rather than prenatal exposure to testosterone. Our experimental design does not allow us to determine whether 2D∶4D ratio differences between offenders and non-offenders is a function of prenatal environment or genetics. However, another line of investigation [30], examining adolescent twins (at ages 11 and 16), reported a strong genetic influence on impulsive tendencies (using self-reported measure).

While genetic factors seem to play a crucial role, our data also illustrate the importance role of education. In line with existing theories [3], offenders exhibited greater impulsive tendencies than nonoffenders. However, education attainment might account for differences in offenders and nonoffenders impulsive tendencies. This is not surprising, as the link between educational attainment and criminal behavior and educational attainment and impulsivity is well founded. One study, for example, looking at the link between education and incarceration has reported that “differences in educational attainment between black and white men explain 23% of the black-white gap in male incarceration rates” ([31], p. 1). Similarly, an examination among smokers [32] has shown that those with less education (no college vs. college education) exhibited greater discounting tendencies. Our current results, however, revealed that 2D∶4D ratio predicted criminality (offenders vs. nonoffenders) even after controlling for individual differences in the education level and impulsivity.

The causal links between 2D∶4D ratio, impulsivity, education, and offending are likely to be highly complex and our current results are less clear in this regard. An inevitable consequence of sampling individuals from different populations (i.e. offenders and nonoffenders), is that they differ in many respects, and this complexity may have clouded our results regarding links between 2D∶4D ratio, impulsivity, and education among offenders and nonoffenders. We hope that the present findings inspire further research in this domain. One fruitful topic that may unpick links involving education would be to measure the 2D∶4D ratio and impulsivity scores of young children as part of a longitudinal investigation of offending.

While it is important to keep these caveats in mind, our analyses suggest that the 2D∶4D ratio measurement may point to an underlying biological basis of criminality through its effects on impulsivity. Our results, however, do not provide a consistent pattern with regard to the exact mediating effects of impulsivity and education on criminality, which would seem to have a more complex relationship with criminal offending.

## References

[1] Anderson DA (1999) The Aggregate Burden of Crime. J Law Econ 42: 611–642.

[2] Binder A (1987) An historical and theoretical introduction. In: Quay H C editor. Handbook of juvenile delinquency. New York: Wiley. pp. 1032

[3] Gottfredson M R, Hirschi TA (1990) General theory of crime. Stanford: Stanford University Press. 297 p.

[4] Barratt ES (1985) Impulsiveness defined within a systems model of personality. In: Speilburger EP, Butcher JN, editors. Advances in personality assessment. Hillsdale: Lawrence Erlbaum Associates. pp. 113–132.

[5] Lynam DR, Miller JD (2004) Personality Pathways to Impulsive Behavior and Their Relations to Deviance: Results from Three Samples. J of Quant Crim 20: 319–341.

[6] McDougall C, Perry AE, Clarbour J, Bowles R, Worthy G (2009) Evaluation of HM Prison Service enhanced thinking skills programme: Report on the outcomes from a randomised controlled trial. Ministry of Justice. Retrieved from http://www.reclassering.nl/documents/Rapport%20ETS.pdf

[7] Barratt ES (1983) The biological basis of impulsiveness: the significance of timing and rhythm disorders. Pers Ind Diff 4: 387–91.

[8] Manning JT, Bundred PE, Newton DJ, Flanagan BF (2003) The second to fourth digit ratio and variation in the androgen receptor gene. Evolu Hum Behav 24: 399–405.

[9] Manning J, Scutt D, Lewisjones D (1998) Developmental stability, ejaculate size, and sperm quality in men. Evolu Hum Behav 19: 273–282.

[10] Martel MM, Gobrogge KL, Breedlove SM, Nigg JT (2008) Masculinized finger-length ratios of boys, but not girls, are associated with attention-deficit/hyperactivity disorder. Behav Neurosci 122 (2) 273–81. 18410167 10.1037/0735-7044.122.2.273 PMC2902868

[11] Lemiere J, Boets B, Danckaerts M (2010) No association between the 2D∶4D fetal testosterone marker and multidimensional attentional abilities in children with ADHD. Dev Med Child Neurol 52: e202–8. 20491856 10.1111/j.1469-8749.2010.03684.x

[12] Stevenson JC, Everson PM, Williams DC, Hipskind G, Grimes M, et al (2007) Attention deficit/hyperactivity disorder (ADHD) symptoms and digit ratios in a college sample. Am J Hum Biol 19 (1)41–50. 17160985 10.1002/ajhb.20571

[13] Blanchard A, Lyons M (2010) An investigation into the relationship between digit length ratio (2D∶ 4D) and psychopathy. The Brit J Foren Prac 12 (2)23–31.

[14] Herschl LC, Highland KB, McChargue DE (2012) Prenatal Exposure to Testosterone Interacts with Lifetime Physical Abuse to Predict Anger Rumination and Cognitive Flexibility among Incarcerated Methamphetamine Users. The Amer J Addic 21: 363–369 doi: 10.1111/j.1521-0391.2012.00246.x 10.1111/j.1521-0391.2012.00246.x 22691016

[15] Aluja A, Garcia LF, Blanch A, De Lorenzo D, Fibla A (2009) Impulsive-disinhibited personality and serotonin transporter gene polymorphism: Association study in an inmate's sample. J of Psyc Res 43: 906–914. 10.1016/j.jpsychires.2008.11.008 19121834

[16] Hönekopp J (2011) Relationships between digit ration 2D∶4D and self-reported aggression and risk taking in an on line study. Pers Ind Diff 51: 77–80.

[17] Coates JM, Herbert J (2008) Endogenous steroids and financial risk taking on a London trading floor. Proc Natl Acad Sci U S A 105: 6167–6172. 18413617 10.1073/pnas.0704025105 PMC2329689

[18] Coates JM, Gurnell M, Rustichini A (2009) Second-to-fourth digit ratio predicts success among high-frequency financial traders. Proc Natl Acad Sci U S A 106: 623–628. 19139402 10.1073/pnas.0810907106 PMC2626753

[19] Roberti JA (2004) A review of behavioral and biological correlates of sensation seeking. J Res Person 38: 256–279.

[20] Eysenck SBG, Eysenck HJ (1978) Impulsiveness and venturesomeness: Their position in a dimensional system of personality description. Psycho Rep 43: 1247–1255. 10.2466/pr0.1978.43.3f.1247 746091

[21] Gordon V, Egan V (2011) What self-report impulsivity measure best predicts criminal convictions and prison breaches of discipline? Psy, Crime Law 17: 305–318.

[22] Aluja A, Garcia LF (2005) Sensation seeking, sexual curiosity and testosterone in inmates. Neuropsychobiology 51: 28–33. 15627810 10.1159/000082852

[23] Sapienza P, Zingales L, Maestripieri D (2009) Gender differences in financial risk aversion and career choices are affected by testosterone. Proc Natl Acad Sci U S A 10.1073/pnas.0907352106. 19706398 10.1073/pnas.0907352106 PMC2741240

[24] Apicella CL, Dreber A, Campbell B, Graye PB, Hoffman M, et al (2008) Testosterone and financial risk preferences. Evolu Hum Behav 29: 384–390.

[25] Pearson M, Schipper BC (2012) The Visible Hand: Finger ratio (2D∶4D) and competitive bidding. Experimental Economics, forthcoming

[26] Roberti JW (2004) A review of behavioral and biological correlates of sensation seeking. J Res Person 38: 256–279.

[27] Bailey AA, Hurd PL (2005) Finger length ratio (2D∶4D) correlates with physical aggression in men but not in women. Biol Psyc 68: 215–222. 10.1016/j.biopsycho.2004.05.001 15620791

[28] Hampson E, Ellis CL, Tenk CM (2008) On the relation between 2D∶4D and sex-dimorphic personality traits. Arch Sex Behav 37: 133–144. 18075733 10.1007/s10508-007-9263-3

[29] Hiraishi K, Sasaki S, Shikishima C, Ando J (2012) The Second to Fourth Digit Ratio (2D∶4D) in a Japanese Twin Sample: Heritability, Prenatal Hormone Transfer, and Association with Sexual Orientation. Arch Sex Behav 41: 711–724 doi:10.1007/s10508-011-9889-z 22270254 10.1007/s10508-011-9889-z

[30] Niv S, Tuvblad C, Raine A, Wang P, Baker LA (2012) Heritability and Longitudinal Stability of Impulsivity in Adolescence. Behav Gene 42: 378–392 doi:10.1007/s10519-011-9518-6 10.1007/s10519-011-9518-6 PMC3351554 22089391

[31] Lochner L, Moretti E (2004) The Effect Of Education On Crime: Evidence From Prison Inmates, Arrests, And Self-Reports. Amer Econ Rev 94: 155–189.

[32] Jaroni JL, Wright SM, Lerman C, Epstein LH (2004) Relationship between education and delay discounting in smokers. Addic Behav 29: 1171–1175. 10.1016/j.addbeh.2004.03.014 15236819

